# Tunable Perfect THz Absorber Based on a Stretchable Ultrathin Carbon-Polymer Bilayer

**DOI:** 10.3390/ma12010143

**Published:** 2019-01-04

**Authors:** Alesia Paddubskaya, Marina Demidenko, Konstantin Batrakov, Gintaras Valušis, Tommi Kaplas, Yuri Svirko, Polina Kuzhir

**Affiliations:** 1 Institute for Nuclear Problems of Belarusian State University, Bobruiskaya 11, 220030 Minsk, Belarus; paddubskaya@gmail.com (A.P.); demidenko@inp.bsu.by (M.D.); kgbatrakov@gmail.com (K.B.); 2 Tomsk State University, 36 Lenin Prospekt, 634050 Tomsk, Russia; 3 Center for Physical Sciences and Technology, Saulėtekio av. 3, LT-10257 Vilnius, Lithuania; gintaras.valusis@ftmc.lt (G.V.); tommi.kaplas@uef.fi (T.K.); 4 Institute of Photonics, University of Eastern Finland, 7 Yliopistokatu, FI-80100 Joensuu, Finland; yuri.svirko@uef.fi; 5 Department of Physics, M.V. Lomonosov Moscow State University, Leninskie Gory, 1199991 Moscow, Russia

**Keywords:** terahertz, perfect absorption, pyrolytic carbon, Salisbury screen

## Abstract

By exploring the Salisbury screen approach, we propose and demonstrate a thin film absorber of terahertz (THz) radiation. The absorber is comprised of a less than 100 nm thick layer of pyrolytic carbon deposited on a stretchable polydimethylsiloxane (PDMS) film followed by the metal film. We demonstrate that being overall less than 200 microns thick, such a sandwich structure absorbs resonantly up to 99.9%of the incident THz radiation, and that the absorption resonance is determined by the polymer thickness, which can be adjusted by stretching.

## 1. Introduction

Growing interest in THz science in the last decade is due to its numerous applications in physics, astronomy, chemistry, biology, and medicine. These include THz imaging, microscopy, non-destructive testing, tomography, medical diagnosis, health monitoring, environmental control, and chemical and biological identification [1,2,3]. Most of these applications involve the detection and decoding of ultra-weak THz signals using bolometers. For example, in astronomy, THz bolometers are key elements of Herschel Space Observatory, James Clerk Maxwell Telescope, and Stratospheric Observatory for Infrared Astronomy [4], which detect the spectra of THz radiation from space objects providing the insights into formation and evolution of galaxies and other fundamental problems of astrophysics. To operate in open space, THz bolometers must be durable and robust, provide detection within a broad frequency range in which the celestial objects may radiate, and comply with payload requirements.

Fabrication of highly sensitive bolometers requires materials and structures with low heat capacity [5] that can absorb almost 100% of the incident radiation. The latter implies perfect matching of the structure impedance with the impedance of free space that can be achieved with bilayers and multi layers comprising of conductive and dielectric films. For example, the perfect absorption can be achieved by placing graphene sheet onto a quarter-wavelength thick dielectric substrate, when radiation comes from the substrate side [6,7]. It is worth noting that graphene (see ref. [8] for review) possesses a record-low heat capacity [9,10,11,12] and short thermal relaxation time [13]. In order to match the impedance of the free space, the graphene sheet can either be placed onto epsilon-near-zero [14] material or on a dielectric film deposited on the top of a metal back reflector. The latter is referred to as the Salisbury screen approach [15,16,17].

To create the Salisbury screen, one needs to deposit a conductive film with a thickness smaller than the skin depth onto a quarter-wavelength thick dielectric layer and to place this sandwich-like structure on a metal support. However, a metallic (e.g., gold, silver or titanium) film should be a few nanometers thick. It will be highly sensitive to defects, aging, oxidation, and surface cleanliness issues [18,19]. It is also impractical to replace the top conductive film with graphene because this requires it her direct deposition [20] or transferring graphene [21] onto the dielectric support. Both techniques are still challenging.

Recently an interesting alternative to graphene for THz and microwave applications has been proposed. It is an ultrathin film of pyrolytic carbon (PyC) [22,23] that can be directly grown on a dielectric or metallic substrate. PyC films, which are comprised of disordered graphene nanoflakes placed in the amorphous carbon host, are robust enough to be transferred, are semitransparent in visible range [24], possess high sheet conductance, and demonstrate a remarkable electromagnetic interference shielding and absorption ability in microwaves and THz [7,25].

As noted in Reference [23], it has been emphasized that *DC* conductivity of PyC is lower than that of graphene (25 × 10^4^ S/m and 100 × 10^4^ S/m, respectively [26,27]). However, it is close to the conductivity of reduced graphene oxide (5.5 × 10^4^ S/m) [28] and is much higher than that of pyrolyzed photoresist (1.6 × 10^4^ S/m) [29]. Thus, by combining thin (<100 nm) PyC film with dielectric layers one can design a flexible and polarization-independent thin film THz absorber.

In this work, we employed the Salisbury screen approach to creating THz absorber based on thin PyC films deposited on polydimethylsiloxane (PDMS) dielectric substrate. Such stretchable, flexible and mechanically robust carbon-polymer bilayer is a removable absorber of variable thickness that can easily stick or be removed from metallic surfaces (including polished ones) without causing any damage. We demonstrate numerically and experimentally that the structure composed of PyC, PDMS and back metal sheet can absorb up to 99.9% of the incident THz radiation and allows one to tune the absorption band by stretching the polymer.

## 2. Theory and Numerical Simulations

**Figure 1:**
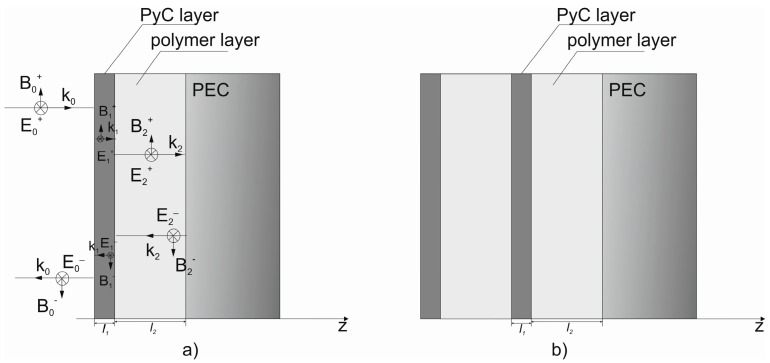
Schematic representation of the wave propagation through multilayer on a metallic substrate, PEC labels perfectly conducting substrate: (**a**) Salisbury screen and (**b**) multilayer structure also known as Jaumann type absorber.

In order to calculate the absorption loss we considered the propagation of the linearly polarized plane wave in the PyC/PDMS sandwich placed on the metal substrate (see Figure 1). The total absorptance of such a multilayer system (often referred to as Gires-Tournois etalon [30]) can be calculated as *A* = 1 − *R*, where *R* is the reflectance. If the substrate is made of a perfect conductor (PEC), the reflectance can be obtained analytically using the continuity of tangential components of the electric and magnetic field sat the vacuum/PyC, PyC/polymer and polymer/PEC interfaces (see Figure 1). At normal incidence, this continuity condition yields:

(1) $$\begin{array}{l} (E_{0}^{+}+E_{0}^{-})-(E_{1}^{+}+E_{1}^{-})=0\\ (E_{0}^{+}-E_{0}^{-})-\sqrt{\varepsilon_{1}}(E_{1}^{+}-E_{1}^{-})=0\\ (E_{1}^{+}\exp(il_{1}k_{1})+E_{1}^{-}\exp(-il_{1}k_{1}))-(E_{2}^{+}\exp(il_{1}k_{2})+E_{2}^{-}\exp(-il_{1}k_{2}))=0\\ \sqrt{\varepsilon_{1}}(E_{1}^{+}\exp(il_{1}k_{1})-E_{1}^{-}\exp(-il_{1}k_{1}))-\sqrt{\varepsilon_{2}}(E_{2}^{+}\exp(il_{1}k_{2})-E_{2}^{-}\exp(-il_{1}k_{2}))=0\\ E_{2}^{+}\exp(i(l_{1}+l_{2})k_{2})+E_{2}^{-}\exp(-i(l_{1}+l_{2})k_{2})=0. \end{array}$$

Here, *l*_1_ and *l*_2_ are the thicknesses of the PyC and dielectric layers. Respectively, $E_{i}^{\pm }$, *k_i_* and *ε_i_* are the amplitudes of the electric field, wavenumber and complex dielectric permittivity in the *i*-th layer. Superscripts “+” and “−” label the forward and backward propagating waves (see Figure 1). The dielectric permittivity of the PyC film is $\varepsilon_{1}\left(\omega \right)=i\sigma_{PyC}/\varepsilon_{0}\omega$, where PyC conductivity $\sigma_{PyC}$ shows a weak frequency dependence in the frequency range of interest [7]. It is worth noting that with no PEC substrate, the last equation in the system (Equation (1)) should be replaced with the following equations:

(2) $$\begin{array}{l} E_{2}^{+}\exp(i(l_{1}+l_{2})k_{2})+E_{2}^{-}\exp(-i(l_{1}+l_{2})k_{2})-{E_{3}}^{+}\exp(-i(l_{1}+l_{2})k_{0})=0\\ \sqrt{\varepsilon_{2}}(E_{2}^{+}\exp(i(l_{1}+l_{2})k_{2})-E_{2}^{-}\exp(-i(l_{1}+l_{2})k_{2}))-{E_{3}}^{+}\exp(-i(l_{1}+l_{2})k_{0})=0, \end{array}$$

where $E_{3}^{+}$ is the complex amplitude of the transmitted wave, *k*_0_ is the free space wavenumber.

**Figure 2:**
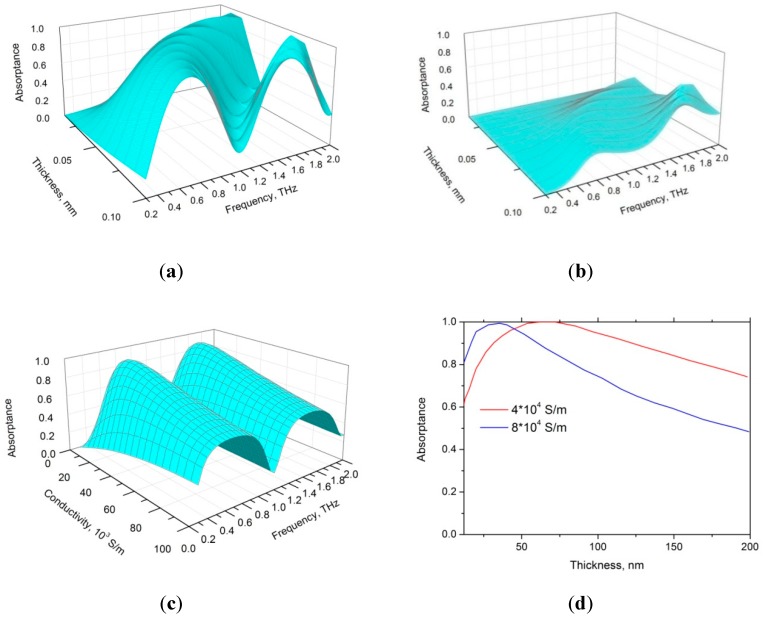

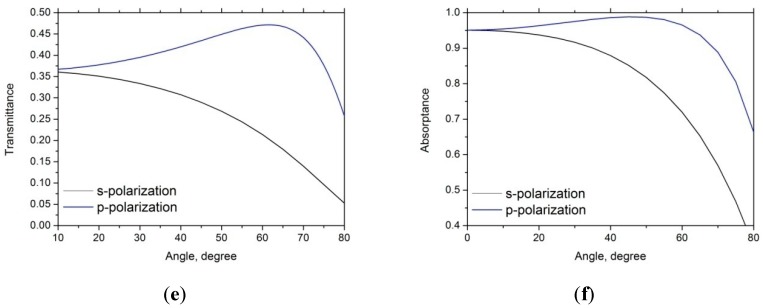
Frequency dependence of absorptance versus thickness of polymer layer calculated using Equation (1) (**a**) with PyC layer and (**b**) without PyC layer at the relative permittivity of polymer of $\varepsilon_{2}$ = 2.4 + 0.14*i*, the thickness of PyC film *l*_1_ = 100 nm and PyC conductivity $\sigma_{PyC}=$ 4 × 10^4^ S/m. (**c**) Absorptance as a function of $\sigma_{PyC}$ at the PyC and polymer layer thicknesses of *l*_1_ = 100 nm and *l*_2_ = 100 μm, respectively. (**d**) The dependence of absorptance on PyC layer thickness (*l*_1_) at the frequency of 0.5 THz and the polymer layer thickness of *l*_2_ = 100 μm. (**e**) Incidence angle dependence of the transmittance of system for the s- and p-polarizations. (**f**) Incidence angle dependence of the PyC/PDMS multilayer absorptance for the s- and p-polarized waves at frequency of 0.5 THz and $\varepsilon_{2}=$ 2.4 + 0.14*i*, $\sigma_{PyC}=$ 4 × 10^4^ S/m, *l*_1_ = 100 nm and *l*_2_ = 100 μm.

The calculated frequency dependence of absorptance $A=1-|E_{0}^{+}/E_{0}^{+}{|}^{2}$ versus the thickness of the polymer layer for samples with and without PyC film and a black reflector is presented in Figure 2a,b.

One can observe from Figure 2a,b that there are Fabry-Perot resonances in reflectance and absorptance spectra of PyC/polymer/PEC and polymer/PEC structures. The resonance frequencies depend on the polymer thickness and refractive index and not on the PyC conductivity. A bare polymer film placed onto PEC surface shows a poor absorption ability while depositing the PyC layer onto the polymer significantly increases (up to 100%) the total absorptance. It is worth noting that the addition of the 100 nm thick PyC film to 100 µm polymer layer left the total thickness virtually unchanged. The coincidence of frequency position of absorption peaks for samples with and without PyC films indicate that multiple reflections govern absorptance of the structure.

In the dependence of the absorptance on the PyC conductivity shown in Figure 2c, the maximum absorptance corresponds to the best possible (at *l*_1_ = 100 nm and *l*_2_ = 100 μm) matching of the structure impedance to the impedance of the free space. Figure 2d shows the dependence of the absorptance on the PyC layer thickness calculated at $\sigma_{PyC}=$ 4 × 10^4^ S/m and $\sigma_{PyC}=$ 8 × 10^4^ S/m. One can observe that for both conductivities there exists the optimum PyC film thickness, which provides the perfect matching of the free space impedance and hence 100% absorptance.

Figure 2e shows the dependence of the PyC/PDMS bilayer transmittance on the incidence angle for *s*- and *p*-polarized waves calculated at the frequency of 0.5 THz for PyC film thickness of 100 nm (compare with our previous work [6]). It is worth noting that the s–polarized wave transmittance decreases with an increase in the incidence angle, while the transmittance of the p-polarized wave transmittance shows non-monotonous dependence on the angle of incidence. Incidence angle dependence of the PyC/PDMS/PEC structure absorptance (see Figure 2d) clearly shows that for both polarizations, such a structure is capable of absorbing more than 90% of the incident radiation provided that the incidence angle does not exceed 50°. This implies that the proposed absorber possesses high angle stability, which is one of the most practical important requirements.

The Jaumann type absorber, which consists of several resistive layers separated by a dielectric (see Figure 1b), is an extension of the single-layer Salisbury screen. It has been demonstrated (for example, see Reference [31]) that the addition of one extra layer makes the absorption bandwidth broader in comparison with that of the single-layer structure. The theoretical analysis of the Jaumann type absorber can be performed by adding continuity conditions for extra interface to Equations (1) and (2).

## 3. Materials and Methods

The PyC films were synthesized on 25 μm thick copper foil (99.8%, Alfa Aesar, Karlsruhe, Germany) by chemical vapor deposition (CVD) in a hot wall tube furnace (30–3000 °C, Carbolite Gero, Neuhausen, Germany). First, the copper foil was loaded into a horizontal furnace, and the system was evacuated to a vacuum for up to 0.06 mBar for 1 h. Then, the substrate was heated up to 1050 °C and annealed for 1 h. The PyC growth was carried out at 1050 °C by introducing the CH_4_ balanced in H_2_ for 30 min. The control over film thickness and roughness was attained by modification of the reaction parameters [22]. In our experiments, the ratio of hydrogen and methane was 5:20 and the total pressure was 20 mBar. After growth, the system was cooled down to room temperature.

**Figure 3:**
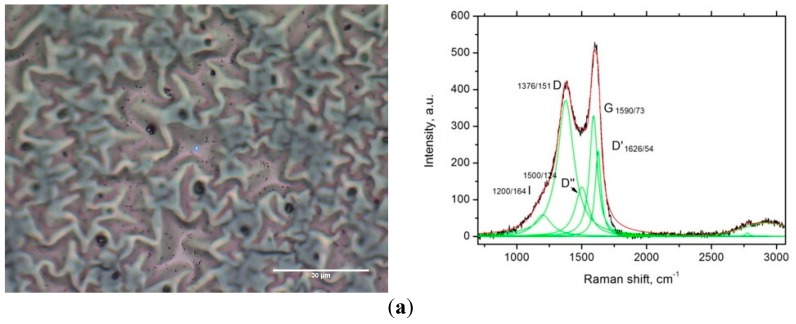

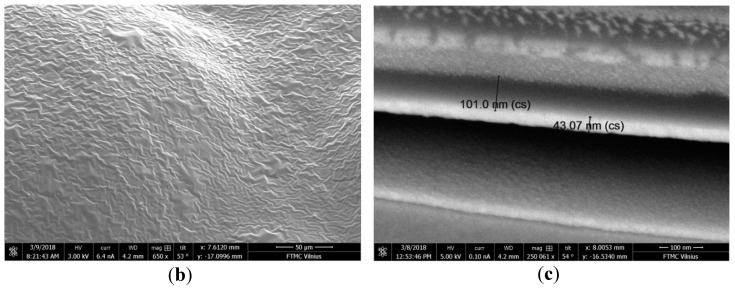
(**a**) The optical image of the surface of the PyC film on the copper substrate and Raman spectra of the PyC film. Fitting (solid green line) shows D (disorder), G (C-C vibration mode), D’ (inter-valley phonon scattering) and D’’ (the localized vibrational modes of the impurities can interact with the extended phonon modes of graphene resulting in the observed splitting) bands [33]. (**b**) SEM image (tilt view 53°) of the PyC film on the top of the copper substrate. (**c**) SEM image of the cross-section PyC film on copper substrate with the deposited ~100 nm thick Pt layer. The Pt layer (top), PyC film (middle, 101 nm), the sputtering copper layer (bottom, 43.07 nm), the cavity between carbon layer and copper foil.

The quality of PyC films was examined by Raman spectrometer combined with the confocal microscope (Nanofinder High End, Tokyo Instruments, Belarus-Japan) with a 600 lines/mm grating and 473 nm laser excitation. Raman spectra (Figure 3a) were collected for samples on a copper substrate using a 50× objective (the optical image is presented in Figure 3a). To reduce sample degradation, the average laser power was kept at 800 μW and exposition time was set to 30 s.

The Raman spectrum of the PyC film in Figure 3a is dominated by two bands centered at 1590 cm^−1^ and 1376 cm^−1^. The former corresponds to the vibration mode of an ideal graphite lattice (first-order G-band ~1580–1590 cm^−1^) and indicates the presence of the crystalline part in the PyCfilm. The latter band is a signature of the graphite disorder [31]. The ratio of intensities of these bands allows on to estimate the average size of graphite crystallites (graphene flakes) [32], which does not exceed 10 nm in the fabricated PyC films. A broad band in the vicinity of 3000 cm^−1^ represents overtones of the one-phonon bands.

Figure 3b shows a scanning electron microscopy (SEM, Helios Nanolab 650, Thermo Fisher Scientific, Hillsboro, OR, USA) image (tilt view) of PyC film synthesized on the copper substrate. To measure the film thickness and investigate the cross-section of the carbon film, a thin Pt layer with a thickness of about 100 nm was deposited on the surface of the selected areas to avoid any damage to the PyC film. The surface was milled using a dual beam-FIB (FEI Helios NanoLab 650, Thermo Fisher Scientific, Hillsboro, OR, USA) and then imaged by the SEM with tilt 54° (see Figure 3c). The contrast difference between Pt/carbon and carbon/copper interfaces indicates that the PyC film thickness varies in the range 80–100 nm over the sample surface. One may also conclude from Figure 3c that the thicker the PyC film, the weaker adhesion that results in film roughness (see Figure 3a).

In order to create the Salisbury screen, we deposited the PyC film on a flexible and stretchable substrate, which was fabricated by mixing the liquid PDMS prepolymer with a cross-linking curing agent at a 10:1 volume ratio (Sylgard 184 silicone elastomer, Dow Corning), pouring into the special form and curing at 80 °C for 15 min. The thickness of the obtained PDMS films was monitored by measuring the THz transmittance spectra.

**Figure 4:**
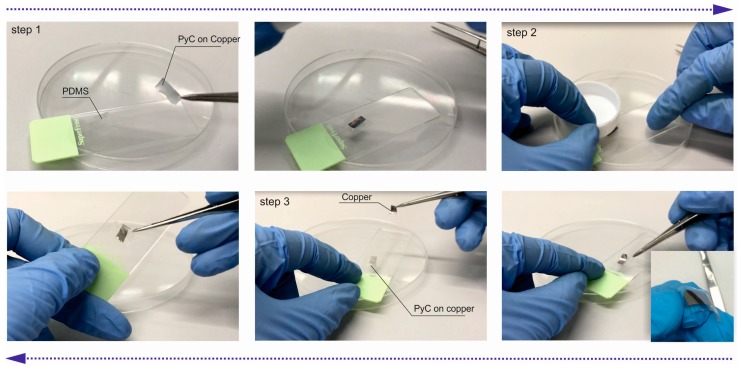
The process of PyC dry transfer to PDMS.

Due to weak adhesion to the copper substrate, the PyC film with a thickness of about 100 nm can be easily transferred onto PDMS using dry transfer technique [34], which is presented in Figure 4. A 1 cm × 1 cm PyC/copper piece was placed PyC-face down on the PDMS film with thickness from 100 up to 200 μm (step 1). The copper foil was gently pressed using a Teflon roller to make the PyC film adhere to the PDMS surface (step 2). The copper foil was easily removed by lifting without significant damage to the PyC film (step 3). The obtained PyC/PDMS flexible assembly was attached to a SiO_2_ substrate with a thin gold film deposit on top (PyC/PDMS/gold sample). In the case of Jaumann type absorber, the procedure could be repeated several times.

## 4. Results

The electromagnetic response of our film in THz frequency range was measured using a commercial THz time-domain spectrometer (T-Spec, EKSPLA, Vilnius, Lithuania), in which THz radiation with frequency up to 2 THz is generated by a photoconductor antenna (low temperature grown GaBiAs) under irradiation with 50–150 fs long laser pulses at wavelength of 1050 nm and power of about 50 mW. The THz pulses were detected by a similar photoconductive antenna. The spectrometer has a module design, i.e., depending on the task it allows one to measure either transmittance or reflectance spectra. The dent helps to put each module exactly in the same place every time. In order to increase the signal to noise ratio, each spectrum was averaged over 1024 measurements. Fast Fourier transform was used to convert the time-domain signal into the frequency domain. To introduce a uniform tensile strain, we used a linear pulse motor.

**Figure 5:**
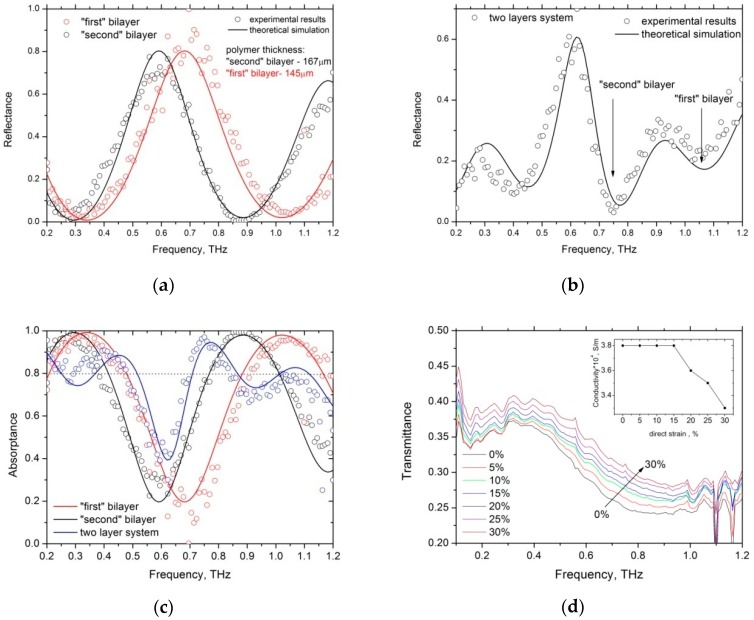
Reflectance (**a**,**b**), and absorptance spectra (**c**) of PyC/PDMS/gold and PyC/PDMS/PyC/PDMS/gold sandwich-like structures, respectively. Numerical calculations were performed by solving Equation (1) with the following material parameters: *l_PyC_* = 100 nm, *l*_PDMS1_ = 145 μm, *l*_PDMS2_ = 167 μm, *σ_PyC_* = 3 × 10^4^ S/m, *ε*_PDMS_ = 2.3 + 0.12i. (**d**) Frequency dependence of transmittance of PyC/PDMS bilayers vs. the applied strain. Inset shows the calculated PyC conductivity as a function of the unidirectional strain.

Figure 5a,b shows the THz reflectance spectra of 145 μm and 167 μm thick PyC/PDMS sandwiches (referred to as “first” and “second” bilayers, respectively) and for combined structure, in which “first” and “second” bilayers are combined into a single multilayer structure. The absorptance spectra for all structures are presented in Figure 5c. The measurement was performed in the frequency range 0.1–1.1 THz that corresponds to the maximum signal-to-noise ratio. One can see that absorptance of PyC/PDMS/gold Salisbury screen exceeds 99.9% at certain frequencies, which depend on the PDMS layer thickness. Figure 5b shows that the addition of the extra bilayer leads to an increase in the total bandwidth while the total absorptance decreases. However, it has been shown in [7] that there exists an optimum combination of the PyC film thickness and the number of the PyC/PDMS bilayers that allow one to achieve simultaneously strong absorption and broad bandwidth.

The thickness of the PyC/PDMS bilayer can be changed by applying tensile strain to the PDMS. Our Raman measurements (the results are not presented here) confirm that stretching of the PDMS does not change the Raman spectrum of the deposited PyC film. Dependence of the PyC/PDMS THz transmittance spectrum on the applied tensile strain is presented in Figure 5d. By comparing our experimental data with the solution of Equation (1) and by considering that the PDMS Poisson ratio is 0.48 [35], we revealed the dependence of the AC conductivity of PyC film on the tensile strain. One can observe from the inset to Figure 5d that $\sigma_{PyC}$ does not change for the strain for up to 15%. At the extension to the original length for up to 30%, the PyC conductivity decreases by about 8% leaving the THz response of the PyC film virtually unchanged. However, the stretching shifts Fabry-Perot resonances because of the decrease in the PDMS thickness, which allows the tuning of the absorptance and transmittance spectra of the PyC/PDMS bilayer.

**Figure 6:**
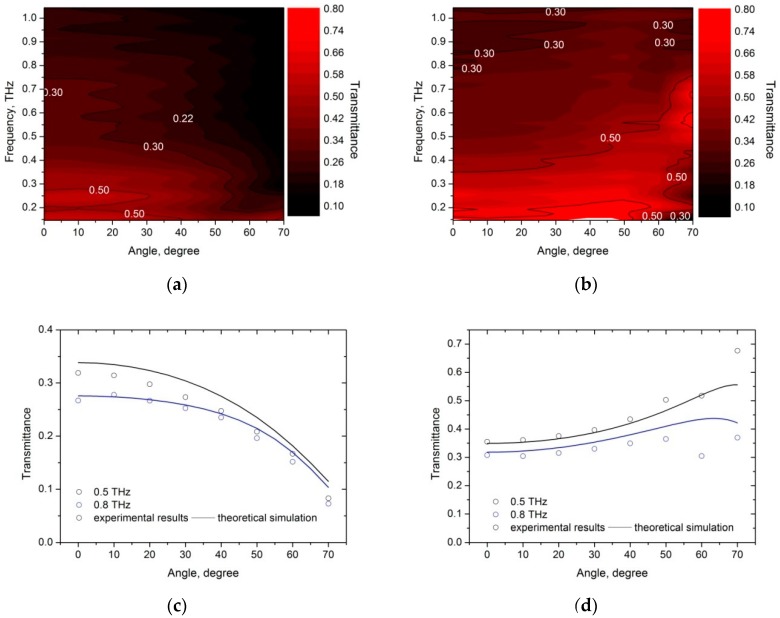
Contour plot of the PyC/PDMS bilayer transmittance for the (**a**) s- and (**b**) p-polarized waves on the frequency/angle of incidence plane (experimental data). The incidence angle dependence of the PyC/PDMS transmittance at 0.5 and 0.8 THz for the (**c**) s- and (**d**) p-polarizations. Circles and solid lines show, respectively, experimental data and results of numerical simulation at *l_PyC_* = 100 nm, *l*_PDMS_ = 200 μm, *σ_PyC_* = 3.2 × 10^4^ S/m, ε_PDMS_ = 2.3 + 0.12i.

Figure 6 shows the measured dependence of the PyC/PDMS bilayer transmittance on the angle of incidence for s- and p-polarized waves in the frequency range 0.1–1.1 THz and comparison of simulation and experimental data at frequencies of 0.5 and 0.8 THz. One can see a good correspondence between experimental and theoretical results. It is also worth noting that this correspondence indirectly confirms a remarkable angle insensitivity of the PyC/PDMS-based Salisbury screen.

## 5. Conclusions

Since the proposed thin sandwich-like structure does not require any patterning or post-processing, and demonstrates the comparable absorptance ability with existing ultrathin THz absorbers [36,37,38,39], our results open an interesting opportunity to fabricate a tunable thin film THz absorber that maintains fabrication simplicity and low cost, has a small thickness and low weight, and offers possibility of integration into the silicon platform. The demonstrated PyC based Salisbury screen possesses a remarkable absorption ability at THz frequencies and can be used as a working element for THz bolometers for aeronautics, as well as in THz transmission imaging, tomography and microscopy systems.
